# Dual task walking in healthy aging: Effects of narrow and wide walking paths

**DOI:** 10.1371/journal.pone.0261647

**Published:** 2021-12-22

**Authors:** Charlotte Hennah, Geraint Ellis, Michail Doumas

**Affiliations:** 1 School of Psychology, Queen’s University Belfast, Belfast, United Kingdom; 2 School of Natural and Built Environment, Queen’s University Belfast, Belfast, United Kingdom; Sao Paulo State University Julio de Mesquita Filho: Universidade Estadual Paulista Julio de Mesquita Filho, BRAZIL

## Abstract

Dual-task walking may lead to gait instability and a higher fall risk in older adults, particularly when walking in a busy city street. Challenging street features such as narrow sidewalks not only discourage walking, but are also likely to be taxing for older adults’ cognitive resources and gait characteristics. The aim of this study was to assess the way older adults’ gait characteristics are affected by walking on a narrow path while performing a challenging cognitive task in lab conditions imitating common urban environments. Nineteen young and eighteen older adults walked on a narrow (40cm) and a wide (80cm) path and performed a cognitive (n-back) task individually adjusted to 80% accuracy. The two tasks were performed separately (Single-Task) and concurrently (Dual-Task). Both groups walked faster, and their step width was narrower on the narrow path. During dual-task walking on the narrow path, older adults showed significant dual-task costs in the cognitive task, gait speed, step width, and stride length. Dual-task walking was associated with decreased gait speed and stride length in both age groups, suggesting that dual-task walking may adversely affect gait, particularly when walking on narrow paths. These conditions may lead to gait instability and an increased fall risk for older adults, particularly when walking along the narrow sidewalks commonly found within the built environment. However, more research is needed in an urban setting to determine the extent of the fall risk narrow sidewalks present for older adults.

## Introduction

Walking is a simple way to increase physical activity for many city-dwelling older adults, bringing numerous health benefits including a lower risk of cardiovascular disease and diabetes [1,2] as well as improving wellbeing and independence [3]. However many elements of the urban environment can make walking challenging for older adults, such as poor street structure with very narrow sidewalks, irregular walking surfaces, and obstacles including trees and parked cars [4]. Such features may lead to greater attentional demands for walking and changes in gait characteristics including decreased speed and increased variability, which have been linked to a higher risk of instability and falls [5,6]. Older adults are particularly susceptible to such gait changes [7] and falls can be detrimental for their quality of life and costly for healthcare systems [3,8]. One street feature that is likely to be challenging for older adults’ gait is sidewalk width.

Regulatory required widths for new sidewalks varies between countries, for example sidewalks in the UK are narrower than those in North America [9,10]. However, narrow sidewalks are commonly found in cities and have the potential to not only create a challenging walking environment for older adults, but also to discourage everyday walking, as pedestrians favor wide paths that do not interrupt foot flow [11,12]. Furthermore, narrow sidewalks disrupt the social element to walking, make it problematic to add amenities (e.g., benches) that usually encourage walking for older adults, and leave little buffer between other pedestrians and traffic. This can present safety concerns which is something older adults have repeatedly described as one of the most critical deterrents to walking [13,14]. Not only could this lead to a reduction in walking and physical activity but walking in these complex urban environments with narrow sidewalks and numerous safety concerns may also require increased levels of attention, particularly for older adults.

Walking is an attentionally demanding task that is often performed while talking, texting or being distracted by other pedestrians. The attentional, or cognitive resource demands of this task have been extensively studied using a dual-task paradigm, comprising of the simultaneous performance of walking and a cognitive task (e.g. counting backwards or n-back) [15]. Evidence from this research suggests that during dual-task walking, performance in both walking characteristics and cognitive tasks is negatively affected, and this effect is reflected in an increase in dual-task costs [16]. This increase has been interpreted using attentional capacity theory [17] suggesting that each task requires a certain amount of cognitive resources. This approach would suggest that young adults should have sufficient resources to perform both tasks and exhibit little to no dual-task costs, however older adults may lack sufficient resources and exhibit greater dual-task costs. However, older adults do not always show greater costs in both tasks. In some cases, for example when balance demands change, they adaptively shift their resources from the cognitive to the motor task to protect their balance and avoid a fall [18–20]. Prioritizing motor or cognitive task performance during dual-task walking has been attributed to a number of factors including the walker’s age, sensorimotor and cognitive capacity, or task difficulty [21,22].

Narrow paths offer a challenging walking environment that many older adults may encounter on a daily basis. However, little is known about the way gait characteristics are affected by dual task walking on such paths. Two previous studies assessed age differences in walking on a narrow and a wide path, but focused on transitions between path widths, rather than on dual-tasking [23,24]. They showed that moving from a wide to a narrow path showed negligible age effects on gait characteristics [23] and that moving from a narrow to a wide path showed that fear of falling affected gait characteristics more than age [24]. Another study assessed dual-task walking on a narrow and a wide path in combination with a visually demanding or a motor demanding task and showed greater dual-task costs in the visual dual task [25]. While this study demonstrated significant age-related dual task costs when walking on a narrow path, the visual and narrow path walking tasks included in Beurskens & Bock’s study both had high visual resource demands. Thus, dual-task interference is likely to have arisen at the perceptual (visual) level, rather than at the level of cognitive resource allocation. Therefore, using an auditory dual-task may be a valuable alternative to visual tasks in dual-task walking studies to reduce perceptual interference.

The aim of this study was to assess age-related differences in gait characteristics during walking on a narrow path, representing a complex element of the built environment, under single- and dual-task conditions using the auditory n-back working memory task [26,27]. The narrow and wide path measurements used were chosen to reflect the common sidewalk widths pedestrians must contend with in cities, and the auditory n-back task was chosen to reduce perceptual interference [28]. Auditory information can be important for walking, particularly for older adults, potentially helping to improve balance [29], increase step length and reduce gait variability [30,31]. However, comparatively, audition is a sensory modality usually less relied upon for walking compared with vision.

Based on previous research [32,33] we predicted an age related dual-task deficit, with young adults showing little to no dual-task costs and little impairment in gait characteristics or cognitive performance, regardless of the complexity of the walking environment, and with older adults’ showing greater dual-task costs in both gait and cognition as task difficulty increases (dual-task walking on a narrow path). Furthermore, in older adults we expected cognitive costs to increase and walking costs to decrease in the more challenging narrow, compared to the wide path condition, reflecting task prioritization. Finally, we expected to see only minor effects of age-related decline in single-task walking performance, in line with previous studies [23,24] and because our older adult group were relatively young and active. However, we expected these differences to emerge as task difficulty (from wide to narrow path) and complexity (from single to dual-task) increased.

## Materials and methods

### Participants

**Fig 1:**
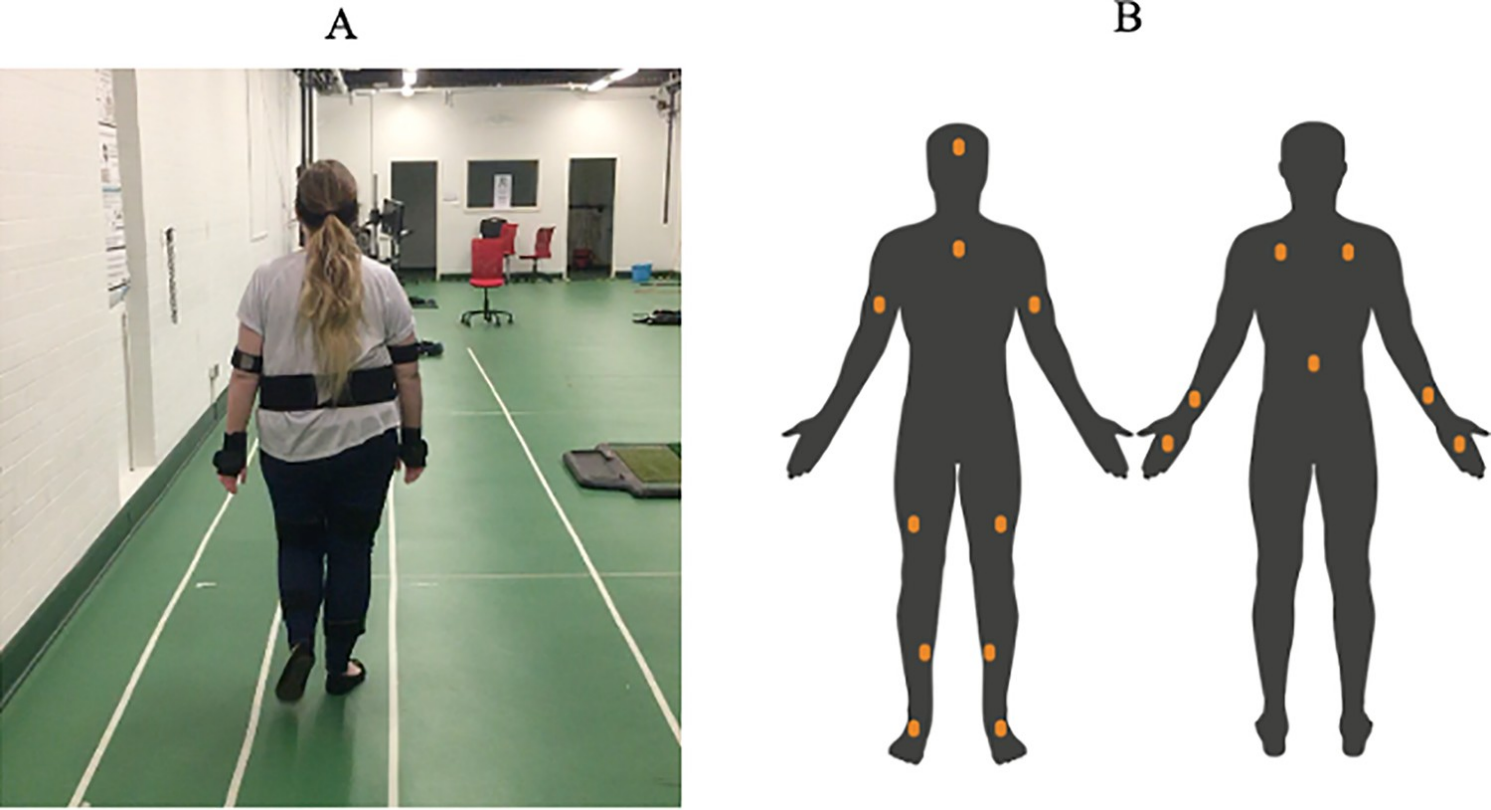
(A) Young adult wearing the xsens sensors walking on the narrow path; and (B) placement position of xsens sensors in the anterior (left) and posterior (right) views of the body, created with BioRender.com.

Using data from a similar dual-task walking study by Nieborowska [34], a power analysis determined that a sample size of 11 participants per group (α = 0.05, β = 0.08) would be necessary to show age and dual task walking effects. Nineteen young (age range: 18 to 35 years, M = 24.9; SD = 5.4, 11 female), and eighteen older adults (age range: 65 to 82 years, M = 69.1; SD = 5.1, 13 female) volunteered to participate in this study. There were no significant differences in average height, weight, or Body Mass Index (BMI) between groups. Participants were recruited via social media, advertising using posters, and directly reaching out to social groups. Young adults were primarily university students, however older participants were primarily recruited from walking or ‘Active Aging’ exercise groups and the local community in general, therefore it is possible our older adult participants may have had a higher level of physical fitness than average for their age group. For both groups inclusion criteria were the ability to walk for five minutes unaided. Although we did not perform a standardized test to determine cognitive ability, training was given to each participant in the n-back task prior to the study, and the level was adjusted depending on their individual performance. Exclusion criteria were the presence of major neurological or musculoskeletal disorders, including those that may affect memory. Participants were also excluded if they had fallen in the previous six months, undergone surgery in the previous year or if they were taking medications that could impair their walking behaviour or memory such as benzodiazepines or antidepressants [35]. As the auditory n-back task is used in this study, it was ensured that all participants were able to hear the stimuli prior to starting the experiment, and sound levels were individually adjusted. No direct incentive was provided for participants to complete this study which was approved by the Faculty of Engineering and Physical Sciences, Queen’s University Belfast Research Ethics Committee (EPS 19_102) and the individual depicted in Fig 1 has given written informed consent (as outlined in PLOS consent form) to publish these case details.

### Tasks and measures

Participants were asked to perform walking and cognitive tasks separately (single-task) and concurrently (dual-task). For the walking tasks two paths were created, a narrow path (width = 40cm) and a wide path (width = 80cm), both 10m in length (Fig 1A). These widths were deemed suitable based on the 100-150cm minimum sidewalk width recommendations for all pedestrians in the UK [9] which considers the requirements for two pedestrians to pass each other comfortably. Our narrow path was narrower than the low end of the range (100cm/2 pedestrians = 50cm) and our wide path was wider than the wide end of the range (150cm/2 pedestrians = 75cm). Participants were asked to stand behind a line drawn 3m behind the start of the 10m path to allow for an acceleration phase, to walk at a comfortable speed to the end of the 10m path and to slow down after that. Masking tape overlaid with hook Velcro outlined the two paths to provide tactile feedback when overstepping. Additionally, photocell sensors (Safety Beam Border Patrol, Lifemax Ltd) were placed at the edges of the tape and gave a loud and unpleasant ‘beep’ if participants overstepped the lines to make overstepping the line similar to stepping off the sidewalk. Overstepping was very rare and the exact number of steps over the edge were not recorded. Gait characteristics were obtained using xsens AWINDA (Xsens Technologies, AWINDA firmware version MTw2 4.4.0), a system that wirelessly captures whole-body movement kinematics in real-time using seventeen wearable Inertial Measurement Unit (IMU) sensors (Fig 1B) which were placed in accordance with xsens manufacturer guidelines [36]. To calibrate the xsens system, we used a standard tape measure to collect height, foot length and additional sole height from the participant. After placing the xsens IMU sensors on the body, participants were asked to walk 5m at a self-selected pace along either the wide or narrow path, then walk back again. This process calibrated xsens and gave a point of origin from which participants would begin walking during the study.

The auditory ‘n-back’ working memory task was used as the cognitive task in single- and dual-task conditions [27,37,38]. Participants were asked to listen to a series of numbers between one and nine, presented through a speaker in a pseudo random order which excluded adjacent number repetition, at fixed intervals using MATLAB with 12 numbers in each series for all conditions. The speaker was stationed in the middle of the room oriented in the participant’s direction and the volume was individually adjusted for each participant, in order for the numbers to be clearly heard. Participants were asked to verbally repeat the numbers one, two or three cycles back (‘1-back’, ‘2-back’, and ‘3-back’ respectively). Numbers were presented while participants sat (Table 1, ST n-back) or walked (Table 1, DT n-back wide/narrow) with an inter-stimulus interval of 1800ms in 12s trials. In dual-task walking trials, participants were asked to wait on the line 3m behind the start of the 10m path, and to start walking when they verbalised the first number (e.g. the fourth presented number in a 3-back trial). The researcher manually recorded the number of correct answers given before an error to determine the participant’s score [20,27].

**Table 1:** Order of presentation of experimental blocks in Single-Task (ST) and Dual-Task (DT) performance. Conditions were counterbalanced, therefore presented to participants either in Order 1 or Order 2.

Block	Context	Tasks	
		Order 1	Order 2
1	ST	N-back	N-back
2	ST	Narrow	Wide
3	ST	Wide	Narrow
4	DT	N-back narrow	N-back wide
5	DT	N-back wide	N-back narrow
6	ST	Narrow	Wide
7	ST	Wide	Narrow
8	ST	N-back	N-back

N-back task difficulty was individually adjusted for each participant to achieve 80% accuracy. Participants were trained in the n-back task and completed three seated trials in each of the 1-back, then 2-back and then 3-back levels of difficulty at 1800ms ISI. Using this titration procedure we determined the difficulty level in which each participant was able to achieve 80% accuracy in this task. This difficulty level was then used in single- and dual-task blocks involving the n-back task (Table 1, Blocks 1, 2, 5 and 8). In the rest of the blocks (Table 1, Blocks 2, 3, 6 and 7) we used a 0-back task in which participants were asked to simply repeat the auditory stimuli to control for possible verbalisation effects.

### Procedure

Prior to arrival in the lab, the Participant Information Sheet was sent via email or read by phone to participants to ensure understanding of the tasks and to confirm eligibility. Participants were also asked to wear clothing suitable for the successful fitting of the xsens AWINDA system. Upon arrival participants provided written informed consent and were afforded the opportunity to ask questions. Then the xsens AWINDA system was set up and calibrated, and participants were encouraged to walk for one minute and familiarise themselves with wearing the sensors.

The experiment started with the titration procedure of the n-back task, seated. Starting at the simplest level ‘1-back’ and potentially progressing through to ‘3-back’, participants completed three-trial blocks in each level of the task. The most challenging level in which they were able to achieve accuracy of 80% was selected to be used in single- and dual-task performance of the n-back task and was considered the participant’s individually adjusted level of performance. In the young adult group, eleven participants used 2-back and eight used 3-back. In the older adult group, two participants used 1-back, twelve used 2-back, and four used 3-back.

After establishing each participant’s n-back level of difficulty, the main experiment started. Experimental conditions were presented in one of the two orders depicted in Table 1, counterbalanced. Each block comprised 3 trials. The first task was ‘ST n-back’, in which participants performed the n-back task at the established 80% level, seated. The next task was one of the two ST walking tasks, in which participants were asked to walk at their preferred pace along the 40cm narrow path (referred to as ‘ST Narrow’) or the 80cm wide path (referred to as ‘ST Wide’ condition). During ST walking trials participants completed the ‘0-back’ task to control for verbalisation thereby ensuring that working memory load was the only additional component between single- and dual-task conditions. Next, dual-task performance was assessed, with participants walking in the two width conditions while simultaneously completing the n-back task at the individually pre-determined level (‘N-back Narrow’ and ‘N-back Wide’ conditions). Finally, the two single task walking conditions were re-assessed to account for improvement or fatigue effects. Participants were offered a two-minute seated break between blocks. Finally, participants completed a seated post-test for the n-back cognitive task, again to account for any performance changes.

### Data analysis

We aimed to study how gait was affected while dual-task walking on a narrow compared to a wide path. To achieve this, gait characteristics during single- and dual-task walking were calculated using data obtained from xsens AWINDA. Full body kinematics were derived from the manufacturer’s MVN Analyse software (version 2019.2.1). We measured each participant’s body dimensions which were added to the software as an input for the full body configuration/model to scale the body segments [39]. Position-time trajectories in three dimensions from the pelvis and the right and left toes were extracted from MVN Analyse and imported into MATLAB 2020b for analysis. Kinematic data in x (anterior-posterior) and y (medio-lateral) coordinates were low pass filtered at 10 Hz using a 4^th^ order Butterworth dual-pass filter, detrended using Principal Component Analysis to align the x axis as determined by xsens software with the direction of the walking path, and finally segmented to ensure that any walking outside the 10m path (acceleration and deceleration phases) was excluded from analysis. Data was recorded from the pelvis, left toe and right toe. Position of the pelvis over time were used to calculate gait speed. Stride length and step time were calculated from the left and right toe segments, where we used the toe-off point to determine step characteristics. Step width was calculated from the distance between left and right toe positions, also at the toe-off point.

Data from ST n-back, ST narrow and ST wide conditions performed early (blocks 1–3) and late in the session (blocks 6–8) were averaged to control for practice or fatigue effects in ST performance.

Finally, we calculated proportional dual-task costs (DTC). In all variables, positive DTC values reflected dual-task costs and negative values reflected dual-task benefits. Specifically, DTCs in individual variables were defined as: reduced accuracy in the n-back task, slower speed, shorter strides, narrower steps and longer step times in dual-, compared with single-task performance. DTCs for the first four variables, in which a lower number reflected a decrease in performance were calculated using the following formula [20]:

DTC=((Single‐task–Dual‐task)/Single‐task)*100

DTCs for step time, for which a higher number reflected a decrease in performance was calculated using this formula:

DTC=((Dual‐task–Single‐task)/Single‐task)*100

Statistical analyses for n-back and all gait characteristics were performed using Mixed-Design analyses of variance (ANOVA) with age (young, older) as between- and task (wide, narrow) and context (single-task, dual-task) as within-subjects factors using Jasp (Version 0.14.1). Dual-task costs were first analysed using a one-sample t-test assessing whether values were significantly different from zero, and then using a Mixed-Design ANOVA with age and width as factors. ANOVAs were followed by simple effects analyses and post-hoc t-tests corrected for multiple comparisons using the Holm-Bonferroni method. Alpha level was set to 0.05.

## Results

### Cognitive

**Fig 2:**
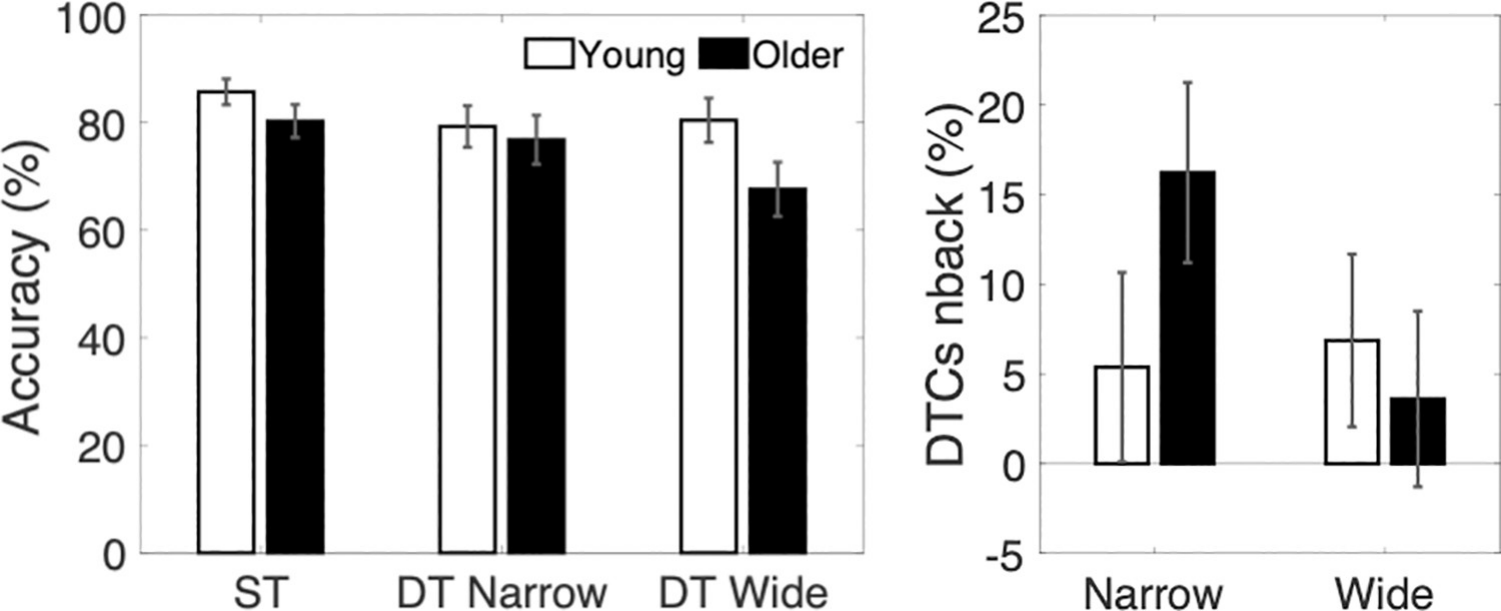
Left panel, average accuracy in the nback task (%) under single- and dual-task conditions. Right panel, cognitive dual-task costs for the young and older group. The asterisk reflects significant DTCs.

Our titration procedure was successful in establishing an individually adjusted single-task accuracy level of approximately 80% for both young and older adults and in eliminating age-differences in single-task n-back performance early in the testing session (Block 1, M_young_ = 84.1%, SD = 9.9; M_older_ = 77.9%, SD = 13.1; p = 0.1). The same task was performed at the end of the experiment (Block 8) to control for improvement or fatigue effects. No group differences were shown at this timepoint either (M_young_ = 88.8%, SD = 15.3; older adults: M_older_ = 82.5%, SD = 20.8; p = 0.3). Data from the two time points were averaged to establish the single-task baseline depicted in Fig 2. A mixed design ANOVA for accuracy in the n-back task in single- and dual-task performance in the two groups (Fig 2) showed no main effect of age (F(1,35) = 1.54, p = 0.22), no main effect of width (F(1,35) = 1.64, p = 0.21), and no interactions. Proportional dual-task costs for working memory accuracy (Fig 2B) were significantly different from zero only for older adults in the narrow condition t(17) = 2.83, p = 0.012.

### Speed

**Fig 3:**
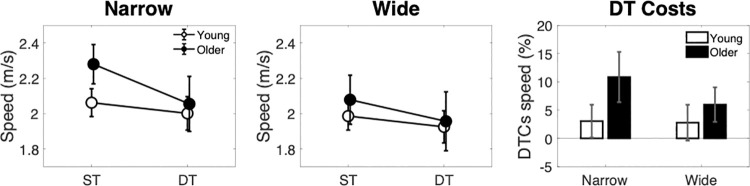
Gait speed across narrow (left panel), wide (middle panel) path conditions, with associated dual-task costs (right panel).

Gait speed results (Fig 3) showed that participants walked faster on the narrow compared with the wide path, as shown by a main effect of width [F(1,33) = 5.62, p = .024, η^2^ = 0.015]. Dual-task performance caused an overall decrease in speed for both groups as shown by a main effect of dual-task context [F(1,33) = 10.68, p = 0.003, η^2^ = 0.015]. No main effect of age or any interactions were shown.

Proportional DTCs (Fig 3) were greater than zero only for older adults in the narrow condition t(16) = 2.41, p = 0.03, reflecting slower walking in dual- compared with single-task conditions in this age group.

### Width

**Fig 4:**
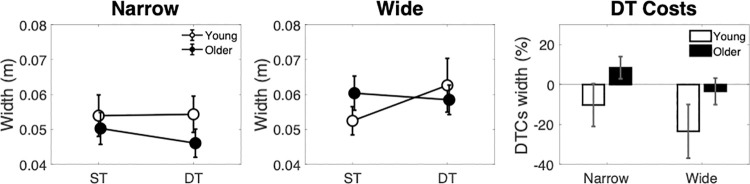
Step width across narrow (left panel), wide (middle panel) path conditions, with associated dual-task costs (right panel).

Step width was significantly narrower in the narrow (Fig 4) compared with the wide condition (Fig 4) as shown by a main effect of width [F(1,31) = 9.783, p = 0.004, η^2^ = 0.032]. No main effects of age or dual-task context were shown, but a context by age interaction [F(1,31) = 4.811, p = 0.036, η^2^ = 0.010] suggested that young adults step width widened, whereas older adults step width narrowed in dual- compared with single-task conditions. However, none of the post-hoc t-tests for this interaction were significant.

No DTCs (Fig 4) were significantly different from zero. However, an ANOVA on these costs showed that older adults’ costs were greater than young adults’ [F(1,34) = 4.384, p = 0.044, η^2^ = 0.129] (Fig 4).

### Stride length

**Fig 5:**
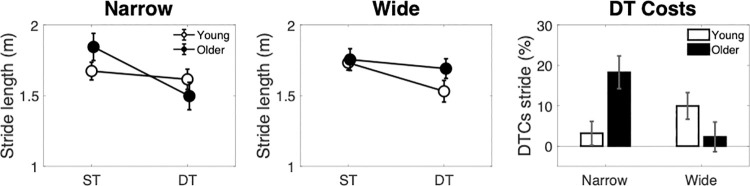
Stride length across narrow (left panel), wide (middle panel) path conditions, with associated dual-task costs (right panel).

In order to obtain an overall measure of stride length we averaged stride length values from the left and right foot (Fig 5). Stride length decreased during dual-task walking as shown by a main effect of dual-task context [F(1,32) = 28.37, p < .001, η^2^ = 0.054], no main effects were found for age or width, however a width by context by group interaction was shown [F(1,32) = 8.78, p = 0.006, η^2^ = 0.027]. Simple effects analyses showed that in the narrow path condition (Fig 5) an overall reduction in stride length in dual- compared with single-task performance was observed as shown by a main effect of dual-task context [F(1,33) = 17.255, p < .001, η^2^ = 0.078], and a context by group interaction [F(1,33) = 8.63, p = 0.006, η^2^ = 0.039]. Post-hoc pair wise comparisons suggested that this reduction in stride length in dual-task performance was only present in the older adult group t(16) = 4.81, p_holm_ < .001. In the wide path (Fig 5), the reduction in stride length in dual compared with single task performance was also present [F(1,33) = 8.06, p = 0.008, η^2^ = 0.037], but no other main effects or interactions were shown.

Dual-task costs in stride length were greater than zero for young adults in the wide path t(17) = 3.02, p = 0.008, and for older adults in the narrow path t(17) = 4.07, p < .001. A mixed-design ANOVA showed a significant Task by Group interaction [F(1,35) = 8.804, p = 0.005, η^2^ = 0.201], reflecting that older adults showed higher dual-task costs on the narrow path, whereas young adults showed greater costs on the wide path. However, none of the post-hoc comparisons for this interaction were significant. No other main effects of interactions were shown.

### Step times

The mean step time for young adults was 366.2ms (SD± 82.5ms) and for older adults 357.1ms (SD± 109.8ms). No significant differences were shown between groups, dual-task context or width conditions and there were no significant dual-task costs in this measure.

## Discussion

The aim of this study was to assess the way older adults’ gait characteristics are affected by walking on a narrow path while performing a demanding cognitive task. Dual-task costs showed that this task altered older adults’ walking, making them walk slower, with narrower gait, and shorter strides, and induced a reduction in accuracy in the cognitive task compared with single-task walking. Furthermore, our results showed that both young and older adults’ walking speed was faster, and their step width was narrower while walking on the narrow path. Our findings suggest that these changes in gait characteristics may also be present while walking in city streets with narrow sidewalks, and are likely to lead to instability and falls especially in older adults [40–43].

We predicted that dual-task costs would increase for older adults as task difficulty increased, and this was confirmed. However, older adults also experienced increased dual-task costs for the walking measures. This is contrary to our prediction that we would see task prioritization in older adults, which would have been reflected in the challenging walking conditions (the narrow path) with improved gait alongside poor cognitive performance [18,20]. If task prioritization were present, then when walking task difficulty increased (from wide to narrow path) dual-task costs would have increased in the cognitive task, as shown in the present study, but they would have decreased or remained the same in the walking task to maintain stability and avoid falling. Instead, increased dual-task costs were observed in both cognitive and walking measures suggesting that there is no evidence for task prioritization here. One reason for this lack of prioritization may be that even our most challenging condition, dual-task walking in the narrow path, was not sufficiently challenging for participants’ balance to induce instability and a fall, therefore they did not need to use cognitive resources to support walking [44]. This could also imply that dual-task walking in a complex urban environment, such as along narrow sidewalks, may not be challenging enough to induce task prioritization either, potentially leaving older adult pedestrians more vulnerable to instability or falling in these environments.

Our results also show that single-task walking on a narrow path is not sufficient to induce age differences in gait characteristics, but these differences emerge in dual-task walking. These results are in line with previous studies on narrow path walking and aging which showed little to no age effects in this task [23,24]. In contrast to these studies, by introducing a walking-cognitive dual-task paradigm, our study showed reliable age effects in narrow path walking, both in terms of the dual-task costs discussed above, but also in individual gait characteristics, namely a shortening in stride length. Our results showing that age-related decline emerged only when walking on the narrow (but not the wide) path when combined with a challenging cognitive task, is in agreement with Beurskens and Bock [25] who showed that dual-task walking on a narrow path and performing a visual task affected older adults’ gait characteristics. However, in their task, vision was used in both the walking and the secondary task and their results can, at least partly, be interpreted as due to perceptual interference in the visual channel. In contrast, in the present study we reduced perceptual interference by using an auditory cognitive task. This has its own limitations however, as audition may be especially influential in older adults’ gait and balance while not affecting younger adults to the same extent [29–31,39]. However, in addition to allowing us to reduce perceptual interference, using an auditory task may allow us to better investigate how older adults walk in similar situations within the built environment, where audition is heavily relied upon. Combined, evidence from our study and by Beurskens and Bock [25] suggest that age-related effects of dual-task walking on a narrow path can emerge, not only at the perceptual level [25] but also at the cognitive resource allocation level.

Older adults showed positive costs in the narrow path condition, however young adults showed significant dual-task costs only in stride length in the wide condition, suggesting that they performed shorter strides in dual- compared with single-task performance only on the wide path. This would suggest young adults’ walking performance worsened on the wide path, contrary to our predictions that gait stability would decrease on the narrow path, and young adults would show no dual-task costs. One interpretation for this effect could be that walking on the wide path in single-task performance was of very low difficulty and required little to no concentration on the cognitive (0-back) or on the walking task. However, when the n-back task was introduced and memory load increased, their concentration and arousal levels increased and as a result they walked with shorter strides. This interpretation also explains the lack of dual-task effects in young adults on the narrow path, and is in line with evidence from postural control suggesting that when a highly demanding cognitive task is introduced young adults reduce their center of pressure movements, which has been attributed to increased concentration or arousal [44].

Our study had a number of limitations. Our sample was a group of very active, healthy older adults recruited from physical activity clubs, with an average age of 69 years, which is a young group of older adults. The low mean age and high physical activity levels of our older adult group are reflected in the lack of main effects of age in any of our measures, particularly as this may have resulted in a group of older adults with higher-than-average physical fitness for their age. These factors could mitigate the dual-task effect on our older adults’ attentional capacity and walking ability, leading to fewer differences between age groups. Nevertheless, the positive dual-task costs and age interactions we observed suggest that even highly active, relatively young older adults show age-related decline when they are asked to perform challenging combinations of walking and cognitive tasks. Another limitation of our study was that the average walking speed was higher than normal, on average 2m/s and close to fast walking in the two groups [45]. One possibility for this high walking speed is the temporal pacing provided by the n-back task stimuli, which were presented at 1800ms intervals, and were present in both single- and dual-task walking trials. Finally, our participants were not cognitively screened using the Mini Mental State Examination or the Montreal Cognitive Assessment test. Use of these tests would have strengthened our sample by excluding older participants with possible scores approaching Mild Cognitive Impairment.

Investigating effects of narrow sidewalk walking is critical in informing our understanding of older adults’ walking behaviour in navigating city streets, to minimise fall accidents and to increase walking for transport in this age group. In this study, participants walked on narrow and wide paths, created using measurements based on common sidewalk widths in UK cities. We correctly predicted that dual-task walking would be challenging for older adults, but less so younger adults, and that the older adult group would show higher dual-task costs on the more complex narrow path compared to the wide path. We also predicted that dual-task walking on the narrow path would produce task-prioritisation in older adults, causing them to prioritize gait and balance, however we did not find evidence for this effect. The task used in this study had high ecological validity because it combined walking on a narrow path while performing a cognitive task, resembling walking on a narrow city sidewalk while engaged in a cognitively taxing activity such as talking on the phone, texting or avoiding obstacles on one’s path. However, to assess walking in a truly ecologically valid setting it is important for future studies to assess dual-task walking in older adults while walking in real and differentiated urban environments. This research is increasingly feasible with the recent advancements in inertial measurement unit (IMU) technology [46,47].

In summary, dual task walking may adversely affect gait in older adults, particularly on a narrow path such as those commonly found in built environments. This has the potential to discourage walking, as well as increase instability and fall risk. This study found that dual task walking in both young and older adults led to reduced gait speed, stride length and width, and dual task walking on the more challenging narrow path exacerbated these effects in older adults, resulting in additional cognitive and gait-related dual task costs. Such gait changes may be indicative of decreased stability, however more research is needed in a real-world setting to determine if narrow sidewalks pose a considerable fall risk to older adults.

## Supporting information

S1 Dataset (XLSX)
